# Psychological well‐being of early and continuously treated phenylketonuria patients

**DOI:** 10.1002/jmd2.12202

**Published:** 2021-02-21

**Authors:** Alena Gerlinde Thiele, Nicole Spieß, Rudolf Ascherl, Maria Arelin, Carmen Rohde, Wieland Kiess, Skadi Beblo

**Affiliations:** ^1^ Hospital for Children and Adolescents, Center for Pediatric Research Leipzig (CPL), Department of Women and Child Health, University Hospital, University of Leipzig Leipzig Germany; ^2^ Present address: Internistisches Therapiezentrum (ITZ) Habichtswald‐Klinik Kassel, Wigandstraße 1, 34131 Kassel Germany

**Keywords:** emotional problems, phenylketonuria, Strengths and Difficulties Questionnaire (SDQ)

## Abstract

**Background:**

Despite enormous advances in therapy, phenylketonuria (PKU) remains an incurable, inherited metabolic disease requiring life‐long treatment with potential to negatively impact quality of life and psychological well‐being. Therefore, the aim of this study was to screen early diagnosed and continuously treated children with PKU on psychological strengths and behavioral difficulties.

**Methods:**

Evaluation of psychological strengths and behavioral difficulties in 49 patients with PKU (23f, 2‐17 years) by Strengths and Difficulties Questionnaire (SDQ; self‐report 11‐17 years and parent‐report 2‐17 years). Comparison to age, sex and BMI‐matched healthy controls (n = 98; 46f).

**Results:**

In patients with PKU and healthy controls median SDQ Total Difficulties Score and median scores of subscales were within the normal range in parent‐ and self‐report, irrespective of sex and age group (children 2‐10 years, adolescents 11‐17 years). No influence of long‐term metabolic control in PKU on SDQ could be revealed. The 2‐ to 10‐year‐old boys with PKU showed significantly higher scores in Prosocial Behavior compared to their healthy peers (*P* = .032). Likewise, adolescent boys with PKU showed fewer Conduct Problems (parent‐report, *P* = .006). Adolescent girls with PKU rated themselves more often as abnormal in the subscale Emotional Problems compared to their healthy peers (*P* = .041). This subscale was also responsible for a significantly different Total SDQ Difficulties Score between patients and their parents' report (*P* = .008).

**Discussion:**

SDQ represents a suitable instrument within the care for patients with PKU. Specific aspects, however, require separate consideration and evaluation with respect to this chronic disease. Special attention should be paid on adolescent PKU girls who seem to be at risk to develop emotional problem.

## Abstract 2

The Strengths and Difficulties Questionnaire is applicable for assessment of psychological wellbeing in patients with phenylketonuria, and reveals scores comparable to healthy peers.

**Abbreviations**
IQR: interquartile range
Phe: phenylalanine
PKU: phenylketonuria
SDQ: Strengths and Difficulties Questionnaire
WSI: Winkler‐Stolzenberg Stratification Index

## 1 INTRODUCTION

Despite enormous advances in therapy, phenylketonuria (PKU; OMIM 261600) remains an incurable, inherited metabolic disease requiring life‐long continuous treatment.^1^ The strict dietary therapy, while successfully preventing the physical and cognitive sequelae, may have a significant negative impact on quality of life and psychological wellbeing of patients.^2^, ^3^ Previous studies reported psychological problems in early diagnosed and treated patients with PKU of different ages, particularly in those with poor metabolic control.^4^, ^5^, ^6^ However, systematic studies on psychological health in patients with PKU are scarce^4^, ^7^, ^8^, ^9^ and former studies might be out of date due to recent developments in PKU therapy.^10^, ^11^ The adherence to therapy and consequently metabolic control show an effect on long‐term outcome concerning executive functions, attention span, working memory as well as depressive symptoms and anxiety.^5^, ^6^, ^12^, ^13^ Identification of relevant psychological strengths and behavioral difficulties in young patients with PKU under continuous treatment is of special interest to recognize requirements in additional psychological care. The aim of this study was to investigate a cohort of early diagnosed and continuously treated children and adolescents with PKU on psychological strengths and behavioral difficulties in comparison to age‐, sex‐ and BMI‐matched healthy controls. Potential influence of age, sex, metabolic control, dietary treatment, and socioeconomic status (SES) was determined.

## 2 PATIENTS AND METHODS

We conducted a monocentric cross‐sectional study in children and adolescents with PKU and mild hyperphenylalaninemia (MHPA) regularly followed‐up in the outpatient clinic for inborn errors of metabolism at the University Hospital for Children and Adolescents, Leipzig. The study was approved by the University of Leipzig's ethics committee (registration number: 440‐12‐17122012) and has been registered with the German Clinical Trial register (DRKS00004942) at the International Clinical Trials Registry Platform.

Inclusion was restricted to patients with PKU and MHPA aged 2 to 17 years who were diagnosed by newborn screening and early and continuously treated according to current national therapy guidelines.^14^ According to pretherapeutic blood Phe concentration, Phe tolerance, and genotype, patients were classified as PKU or MHPA. Patients with a BH_4_‐sensitive PKU had to be on BH_4_ treatment for at least 1 year prior enrolment to ensure adaption to new therapy outline and dietary consolidation.^15^, ^16^ Metabolic control was assessed by regular determination of Phe in dried blood samples. Phe intake of patients on diet was calculated from detailed diet records over 3 days at least twice yearly, which provided exact weight of all consumed foods. Late diagnosed patients or patients with additional diseases were excluded from the analysis.

The control group, matched by age, sex, and BMI,^17^ consisted of participants of the “Leipzig Research Center for Civilization Diseases” (LIFE) Child Study.^18^ Since some controls who could be exactly matched by all three variables had partially, and nonoverlapping missing questionnaires, we decided to include two controls per PKU patient.

### 2.1 Strengths and Difficulties Questionnaire

The Strengths and Difficulties Questionnaire (SDQ) is a well‐established validated screening tool assessing psychological strengths and behavioral difficulties in children and adolescents aged 2 to 17 years.^19^, ^20^ Two versions of the SDQ were used: self‐report for adolescents aged 11 to 17 years and a parent‐report for children aged 2 to 17 years. The SDQ comprises 5 scales with 5 items per scale. Every item represents a statement about a negative or positive attribute and can be answered on a 3‐point Likert scale (“not true,” “somewhat true,” or certainly true”).^19^

While the scale “*Pro‐Social Behavior*” is assessed as a psychological strength, the other 4 scales (“*Emotional Symptoms*,” “*Conduct Problems*,” “*Hyperactivity*,” “*Peer Problems*”) reflect behavioral difficulties.^19^ A Total Difficulties score can be calculated from the latter 4 scales. The Total Difficulties score (0‐40 points) as well as the calculated score of each single scale (0‐10 points) can be assigned to the categories “normal,” “borderline,” and “abnormal” (Table 4).^19^, ^20^ Higher scores express more difficulties. In contrast, in the scale Prosocial Behavior, a higher score represents a pronounced psychological strength. The extended version used here, includes further questions about mood, concentration, behavior, and interaction with other persons^21^ to assess the impact of abnormalities in the SDQ result on everyday life. Duration of problems, subjectively experienced burden, social impairment (at home, with friends in school or leisure time) and the resulting burden on relatives are assessed as single items and then transformed into the “*Impact Score*.” Again, a three point Likert Scale is used for evaluating the items (“not at all”/”hardly,” “clear,” and “heavy”), and the score is assigned to the categories “normal” (score 0), “borderline” (score 1) or “abnormal” (score 2‐10).^21^, ^22^

### 2.2 Current therapy and metabolic control

Data on current therapy (eg, Phe‐restricted diet or BH_4_ supplementation) and biochemical parameters were gathered from electronic medical records. All patients provide weighed diet records over three consecutive days at least twice a year. These data were used to determine current Phe‐intake. To assess long‐term metabolic control, all dried blood Phe concentrations 1 year prior to study enrollment were analyzed. Additionally, dried blood Phe concentration was measured at the study visit. Phe and tyrosine concentrations in dried blood were determined by liquid chromatography/tandem mass spectrometry as previously described.^23^

### 2.3 Socioeconomic status

To classify patients according to their socioeconomic status (SES), the Winkler‐Stolzenberg (WSI) Stratification Index^24^ was used. This multidimensional tool combines information household income, parental educational level, and occupational status. A score ranging from 1 to 7 is assigned to each of the three indicators according to seven possible answers per indicator. The Winkler Index (range 3‐21 points) is calculated by addition of these three scores. This score is categorized into three status groups corresponding to low (score 3‐8), middle (9‐14), and high (15‐21) SES.^25^

### 2.4 Data analysis

Data are presented as median and interquartile range (IQR). Since all data referring to the core question of the study were not normally distributed nonparametric statistics were performed by Wilcoxon‐test for paired samples, Mann‐Whitney‐*U*‐test for two independent samples, and Kruskal‐Wallis‐test for more than two independent samples, respectively. Since the main focus of our study was to reveal qualitative differences between patients with PKU and their healthy peers, and numerical results made clear that no major group differences could be expected, we chose not to introduce adjustment in the analytical statistics. Categorical variables (“normal,” “borderline,” “abnormal”) were analyzed by using cross tables and Fisher's exact test. Relationship between two variables was assessed by using the Spearman correlation. The Cohen classification was used to interpret the calculated correlation (weak effect for *r* = .1, medium effect for *r* = .3, and strong effect for *r* = .5). Weighting was not applied. All statistical procedures were performed with IBM SPSS Statistics Version 23 for Windows. A nominal level of *P* < .05 was regarded as statistically significant.

## 3 RESULTS

### 3.1 Patient characteristics

Of 62 patients with PKU screened at the outpatient clinic, 52 met the inclusion criteria and 49 participated in the study. While 30 of these patients followed a Phe‐restricted diet, the remaining 19 patients were off‐diet due to either BH_4_‐treatment (n = 7) or a diagnosis of MHPA (n = 12, children n = 10, adolescents n = 9). The control group consisted of 98 metabolically healthy children and adolescents from the LIFE Child study. The characteristics of patients and controls are shown in Table 1.

**TABLE 1:** Characteristics of patients and controls

	Patients (total)	Classical PKU	MHPA	BH_4_‐sensitive PKU	Controls	*P*
Number (n)	49	25	10	14	98	
Gender						
Male (n)	26	13	5	8	52	1.000^a^
Female (n)	23	12	5	6	46	
Age (years) Median [IQR]	9 [4.5; 14.0]	8 [3.5; 13.5]	8.5 [3.0; 15.3]	11.5 [6.8; 14.3]	10 [4.0; 13.3]	.768^a^
BMI (kg/m^2^) Median [IQR]	17.7 [16.2; 19.0]	17.1 [16.0; 18.8]	17.4 [16.2; 20.6]	18.3 [16.9; 19.9]	17.9 [16.1; 19.7]	.763^a^
WSI‐Index^b^ Median [IQR]	13 [10.0; 15.0]	12 [9.0; 14.0]	12.5 [6.0; 14.5]	13 [11.8; 19]	14 [11.3; 16.0]^e^	**.046** ^a^
Median mean dried blood Phe concentration (μmol/L) at study visit [IQR]	213 [152; 398]	306 [138; 499]	207 [141; 212]	224 [173; 365]	n. a.	.233
Median mean dried blood Phe concentration (μmol/L) 1 year prior enrollment [IQR]	231 [199; 437]	250 [215; 577]	186 [163; 213]	261 [217; 360]	n. a.	**.003** ^c^
Median Phe tolerance^f^ (mg/day) **[**IQR]	450^g^ [339; 1000]	400^h^ [260; 480]	n. a.	1170^i^ [1000; 1200]	n. a	**<.001** ^d^

*Note*: The *P* values in bold letters represent significant differences (*P* < .05).

Abbreviations: MHPA, mild hyperphenylalaninemia; n. a, not applicable; PKU, phenylketonuria.

^a^ Comparison between patients (total) vs controls, Mann‐Whitney‐*U*‐test.

^b^ WSI stratification Index: lower class 3‐8, middle class 9‐14, upper class 15‐21.

^c^ Comparison between patients with classical PKU vs BH_4_‐sensitive PKU vs MHPA, Kruskal‐Wallis‐test.

^d^ Comparison between patients with classical PKU vs BH_4_‐sensitive PKU, Mann‐Whitney‐*U*‐test.

^e^ n = 88.

^f^ Applicable for PKU patients on diet.

^g^ n = 30.

^h^ n = 23.

^i^ n = 7.

Median mean Phe concentrations at the study visit as well as long‐term metabolic control were within the age‐specific therapeutic ranges. Patients with MHPA exhibited significantly lower long‐term mean Phe concentrations compared to patients with classical PKU (*P* = .005) and patients with BH_4_‐sensitive PKU (*P* = .021).

Regarding SES, represented by the median WSI Stratification Index, both groups (patients and controls) could be assigned to the middle class, with a slightly but significantly higher median score of the control group.

### 3.2 SDQ parent‐report

The SDQ parent‐report was analyzed according to the represented age group (2‐10 years, referred to as children and 11‐17 years, referred to as adolescents). The results are presented in Tables 2 and 3.

**TABLE 2:** Comparison of SDQ total difficulties score and subscale for patients and controls from parent‐ and self‐report: Median Total Difficulties Score and median scores of subscales were within the normal range in all investigated groups. This is true for parent‐ and self‐report. Specific differences in subscales can be read from the table

	Patients with PKU			Controls							
	Parent‐report		Self‐report	Parent‐report		Self‐report					
	2‐10 years	11‐17 years	11‐17 years	2‐10 years	11‐17 years	11‐17 years					
	n = 27	n = 22	n = 22	n = 52	n = 36	n = 42					
	(12f, 15 m)	(11f, 11 m)	(11f,11 m)	(20f, 32 m)	(20f, 16 m)	(23f, 19 m)	*P* ^1^ *	*P* ^2^ *	*P* ^3^	*P* ^4^ *	*P* ^5^ *
Total Difficulties Score											
Median [IQR], female	8.0 [4.0; 13.8]	**7.0** [4.0; 9.0]	**8.0** [7.0;17.0]	8.5 [5.0; 13.8]	7.5 [3.0; 13.8]	10.0 [7.0; 12.0]	.737	.617	.750	**.008**	.154
Median [IQR], male	9.0 [6.0; 12.0]	6.0 [3.0; 9.0]	8.0 [6.0;12.0]	10.5 [7.0; 14.0]	5.5 [4.0; 9.8]	6.0 [4.0; 11.0]	.530	.855	.387	.117	.772
Median mean dried blood Phe concentration (μmol/l) at study visit r [p]	0.189 [0.345]	−0.022 [0.924]	−0.030 [0.896]	n. a.	n. a.	n. a.					
Median mean dried blood Phe concentration 1 year prior enrollment r [p]	0.094 [0.641]	−0.090 [0.689]	−0.073 [0.747]	n. a.	n. a.	n. a.					
Emotional Problems											
Median [IQR], female	2.0 [0.0; 3.8]	2.0 [1.0; 4.0]	3.0 [2.0; 7.0]	2.0 [1.0; 3.0]	2.0 [1.0; 4.0]	3.0 [2.0; 5.0]	.867	.715	.499	.055	.179
Median [IQR], male	0.0 [0.0; 2.0]	2.0 [0.0; 3.0]	1.0 [0.0; 3.0]	1.0 [0.0; 3.0]	0.5 [0.0; 2.0]	1.0 [0.0; 2.0]	.419	.324	.647	.727	.598
Hyperactivity											
Median [IQR], female	2.5 [1.0; 5.5]	2.0 [0.0; 3.0]	2.0 [1.0; 6.0]	4.0 [1.3; 5.0]	3.0 [1.0; 4.0]	3.0 [1.0; 4.0]	.619	.446	.964	.090	.405
Median [IQR], male	4.0 [3.0; 5.0]	2.0 [2.0; 5.0]	3.0 [1.0; 6.0]	5.0 [3.0; 6.0]	2.5 [2.0; 4.8]	3.0 [1.0; 4.0]	.320	.953	.659	.684	.682
Conduct Problems											
Median [IQR], female	2.0 [0.3; 3.8]	1.0 [0.0; 2.0]	1.0 [0.0; 4.0]	2.0 [1.0; 3.8]	2.0 [0.0; 3.0]	1.0 [1.0; 2.0]	.828	.228	.845	.250	.465
Median [IQR], male	3.0 [2.0; 4.0]	**0.0** [0.0; 1.0]	1.0 [0.0; 3.0]	3.0 [1.0; 4.0]	**1.0** [1.0; 2.0]	1.0 [0.0; 2.0]	.983	**.006**	.680	.164	.429
Peer Problems											
Median [IQR], female	1.5 [0.3; 2.0]	0.0 [0.0; 2.0]	2.0 [1.0; 3.0]	1.0 [0.0; 2.0]	**1.0** [0.0; 2.8]	**2.0** [1.0; 3.0]	.822	.360	.719	.141	**.022**
Median [IQR], male	1.0 [0.0; 1.0]	1.0 [0.0; 3.0]	2.0 [1.0; 3.0]	1.0 [0.0; 3.0]	0.5 [0.0; 1.8]	2.0 [1.0; 3.0]	.271	.708	.749	.375	.453
Prosocial Behavior											
Median [IQR], female	8.0 [7.0; 9.0]	10.0 [7.0; 10.0]	8.0 [8.0; 9.0]	8.0 [6.3; 9.0]	9.0 [8.0; 9.8]	9.0 [8.0; 9.0]	.520	.474	.255	.176	.992
Median [IQR], male	**8.0** [7.0; 9.0**]**	8.0 [7.0; 10.0]	9.0 [7.0; 9.0]	**7.0** [6.0; 9.0]	8.5 [7.0; 9.8]	7.0 [6.0; 10.0]	**.032**	.849	.379	.711	.141

*Note*: P^1‐3^ Mann‐Whitney‐*U*‐test; *P* ^4‐5^ Wilcoxon‐test; *P* ^1^ age group 2‐10 years patients vs controls, parent‐report; *P* ^2^ age group 11‐17 patients vs controls, parent‐report; *P* ^3^ age group 11‐17 patients vs controls, self‐report; *P* ^4^ Comparison parent‐report vs self‐report age group 11‐17 of patients with PKU; *P* ^5^ Comparison parent‐report vs self‐report age group 11‐17 of controls.

Abbreviations: n. a, not applicable; PKU, phenylketonuria; SDQ, Strengths and Difficulties Questionnaire.

^*^ *P* values in bold numbers represent significant differences *P* < .05, for better visualization the corresponding results are also displayed in bold numbers.

**TABLE 3:** Comparison of total difficulties score and subscales: Percentages of patients and controls from parent‐ and self‐report with a borderline or an abnormal score. Comparing self‐report of patients and controls revealed significant differences within the subscales *“Emotional problems”* and *“Hyperactivity”*

	Patients with PKU			Controls							
	Parent‐report		Self‐report	Parent‐report		Self‐report					
	2‐10 years	11‐17 years	11‐17 years	2‐10 years	11‐17 years	11‐17 years	*P* ^1^ *	*P* ^2^ *	*P* ^3^ *	*P* ^4^	*P* ^5^
	n = 27	n = 22	n = 22	n = 52	n = 36	n = 42					
	(12f, 15 m)	(11f, 11 m)	(11f, 11 m)	(20f, 32 m)	(20f, 16 m)	(23f, 19 m)					
Total Difficulties Score											
Borderline (%/n), female	8.3/1	9.1/1	18.2/2	15.0/3	15.0/3	8.7/2	1.000	1.000	.567	.586	1.000
Borderline (%/n), male	6.7/1	0.0/0	0.0/0	12.5/4	0.0/0	5.3/1	.645	.645	1.000	n. s.	n. s.
Abnormal (%/n), female	16.7/2	0.0/0	9.1/1	10.0/2	10.0/2	0.0/0	1.000	.516	.300	.474	1.000
Abnormal (%/n), male	6.7/1	0.0/0	0.0/0	15.6/5	6.3/1	0.0/0	.645	1.000	n. s.	n. s.	1.000
Emotional Problems											
Borderline (%/n), female	8.3/1	18.2/2	0.0/0	10.0/2	10.0/2	17.4/4	1.000	.602	.550	.471	.591
Borderline (%/n), male	0.0/0	0.0/0	0.0/0	15.6/5	12.5/2	0.0/0	.301	.499	n. s.	n. s.	.483
Abnormal (%/n), female	16.7/2	18.2/2	**27.3/3**	0.0/0	20.0/4	**0.0/0**	.135	1.000	**.041**	1.000	.227
Abnormal (%/n), male	13.3/2	0.0/0	0.0/0	3.1/1	0.0/0	0.0/0	.287	n. s.	n. s.	n. s.	n. s.
Hyperactivity											
Borderline (%/n), female	8.3/1	0.0/0	18.2/2	5.0/1	0.0/0	0.0/0	1.000	n. s.	.085	.214	n. s.
Borderline (%/n), male	6.7/1	0.0/0	**27.3/3**	12.5/4	0.0/0	**0.0/0**	.645	n. s.	.050	.214	n. s.
Abnormal (%/n), female	16.7/2	0.0/0	9.1/1	10.0/2	5.0/1	0.0/0	.611	1.000	.281	.450	1.000
Abnormal (%/n), male	6.7/1	9.1/1	0.0/0	15.6/5	0.0/0	10.5/2	.645	.407	1.000	1.000	1.000
Conduct Problems											
Borderline (%/n), female	8.3/1	0.0/0	9.1/1	15.0/3	20.0/4	4.3/1	1.000	.268	.490	.450	.338
Borderline (%/n), male	33.3/5	0.0/0	0.0/0	31.3/10	12.5/2	0.0/0	1.000	.500	n. s.	n. s.	.224
Abnormal (%/n), female	25.0/3	0.0/0	18.2/2	25.0/5	10.0/2	0.0/0	1.000	.499	.091	.214	.469
Abnormal (%/n), male	26.7/4	9.1/1	9.1/1	31.3/10	6.3/1	5.3/1	1.000	1.000	1.000	1.000	.464
Peer Problems											
Borderline (%/n), female	8.3/1	0.0/0	18.2/2	5.0/1	10.0/2	4.3/1	1.000	.520	.252	.479	1.000
Borderline (%/n), male	6.7/1	18.2/2	0.0/0	6.3/2	0.0/0	5.3/1	1.000	.142	1.000	.189	1.000
Abnormal (%/n), female	0.0/0	18.2/2	0.0/0	15.0/3	20.0/4	4.3/1	.279	1.000	1.000	.479	.335
Abnormal (%/n), male	0.0/0	9.1/1	0.0/0	21.9/7	12.5/2	0.0/0	.078	1.000	n. s.	.450	.483
Prosocial Behavior											
Borderline (%/n), female	0.0/0	9.1/1	9.1/1	5.0/1	5.0/1	4.3/1	1.000	1.000	.534	1.000	1.000
Borderline (%/n), male	0.0/0	9.1/1	0.0/0	15.0/3	6.3/1	15.8/3	.541	1.000	.532	1.000	.598
Abnormal (%/n), female	0.0/0	0.0/0	9.1/1	5.0/1	0.0/0	0.0/0	1.000	n. s.	.323	1.000	n. s.
Abnormal (%/n), male	0.0/0	0.0/0	9.1/1	12.5/4	0.0/0	0.0/0	.282	n. s.	.393	1.000	n. s.

*Note*: P^1^ age group 2‐10 years patients vs controls, parent‐report; *P* ^2^ age group 11‐17 patients vs controls, parent‐report; *P* ^3^ age group 11‐17 patients vs controls, self‐report; *P* ^4^ Comparison parent‐report vs self‐report age group 11‐17 of patients with PKU; *P* ^5^ Comparison parent‐report vs self‐report age group 11‐17 of controls.

Abbreviations: n. s., not significant; n, absolute amount of patients with PKU/controls.

^*^ *P* values in bold numbers represent significant differences *P* < .05, for better visualization the corresponding results are also displayed in bold numbers, Fisher's exact test.

### 3.3 Children with PKU (SDQ parent‐report)

Irrespective of sex, median Total Difficulties Score and median scores of subscales were within the normal range. The only exception represents the subscale “Conduct Problems” in which the 2‐10‐year‐old boys revealed a borderline median score as reported by the parents. Interestingly, median score for "Prosocial Behavior" was in the upper normal range in this group and none of the included patients showed a borderline or abnormal result.

### 3.4 Adolescents with PKU (SDQ parent‐report)

In adolescents with PKU, median Total Difficulties Score and median scores of subscales were within the normal range. However, some individual patients, especially adolescent girls, reached borderline scores in the Total Difficulties Score and borderline and abnormal scores in some subscales. For the parents, “Emotional Problems” were the main focus for their daughters. In male adolescents, the highest percentage of borderline or abnormal scores was found in the subscale “Peer Problems”.

### 3.5 Adolescents with PKU: SDQ self‐report and comparison to parent‐report

As revealed by SDQ self‐report, adolescent girls and boys showed normal median Total Difficulties Score and normal median scores in all subscales. However, their median Total Difficulties Score was higher compared to the respective parent‐report. For girls, this difference was significant (*P* = .008). Adolescent girls more often rated themselves as abnormal in the subscale “Emotional Problems” compared to their parents (27.3% vs 18.2%).

### 3.6 Comparison of patients and controls

Comparison of children and adolescents with PKU and metabolically healthy controls revealed no significant differences, neither in the median of the SDQ Total Difficulties Score nor in its four subscales. The overall rating‐profile of patients and controls was very similar, pointing out equal areas of strengths and difficulties. This was true for the parent‐reports as well as for the self‐report.

However, 2‐ to 10‐year‐old boys with PKU showed a significantly better "Prosocial Behavior" compared to their healthy peers (*P* = .032). In addition, adolescent boys with PKU showed a lower score for "Conduct Problems" in comparison to their healthy controls (*P* = .006).

During adolescence, a significantly higher percentage of girls with PKU showed abnormal results in the subscale "Emotional Problems" compared to healthy adolescent girls (27% vs 0%, *P* = .041, Tables 3 and 4) in the self‐report. In contrast, none of the adolescent boys rated themselves as borderline or abnormal in this subscale, whether suffering from PKU or not.

**TABLE 4:** Reference values of SDQ parent‐ and self‐report^19^, ^20^

	SDQ Parent‐report			SDQ Self‐report		
Subscale	Normal	Borderline	Abnormal	Normal	Borderline	Abnormal
Total Difficulties Score	0‐13	14‐16	17‐40	0‐15	16‐19	20‐40
Emotional Problems	0‐3	4	5‐10	0‐5	6	7‐10
Hyperactivity	0‐5	6	7‐10	0‐5	6	7‐10
Conduct Problems	0‐2	3	4‐10	0‐3	4	5‐10
Peer Problems	0‐2	3	4‐10	0‐3	4‐5	6‐10
Prosocial Behavior	6‐10	5	0‐4	6‐10	5	0‐4

Abbreviation: SDQ, Strengths and Difficulties Questionnaire.

### 3.7 Impact score

The median Impact Score correlated positively with the Total Difficulties Score and the scores of all subscales (Table 5). As a consequence, an abnormal impact score could be revealed in SDQ‐subscales with abnormal results. This was especially prominent in the parent‐report for adolescent girls where the subscale "Emotional Problems" showed a strong correlation with the Impact Score (patients: *r* = .678, *P* = .022, controls: *r* = .720, *P* < .001). This was also true for the adolescent self‐report in girls with PKU (*r* = .850, *P* = .001) and in the parent‐report for 2‐ to 10‐year old boys with PKU (*r* = .383, *P* = .031).

### 3.8 Influence of diet and metabolic control on SDQ

The median Total Difficulties as well as median scores of all subscales were within the normal range (Table 6) in all patient groups, irrespective of following a Phe restricted diet or not. However, adolescents off diet scored significantly higher in the subscale *“Hyperactivity”* in comparison to their counterparts on diet (Table 6).

**TABLE 5:** Correlation of Impact Score and Total Difficulties Score: the median Impact Score was within the normal range by SDQ parent‐ and self‐report irrespective of suffering from PKU and it correlated positively with the Total Difficulties Score

	Patients with PKU			Controls		
	Parent‐report		Self‐report	Parent‐report		Self‐report
	2‐10 years	11‐17 years	11‐17 years	2‐10 years	11‐17 years	11‐17 years
	n = 27	n = 22	n = 22	n = 52	n = 36	n = 42
Total Difficulties Score; Median [IQR]	9.0 [5.0; 12.0]	7.0 [3.8; 9.0]	8.0 [6.8; 12.8]	10.0 [6.0; 14.0]	6.0 [3.3; 12.0]	8.0 [6.0; 12.0]
Impact Score, Median [IQR] Maximum	0.0 [0.0; 0.0] 4.0	0.0 [0.0; 0.0]^a^ 6.0	0.0 [0.0; 1.0] 4.0	0.0 [0.0; 0.0] 8.0	0.0 [0.0; 1.0]^b^ 9.0	0.0 [0.0; 0.0] 2.0
*r*	**.423**	**.543**	**.595**	**.437**	**.532**	.266
*P*	.028	.011	.003	.001	<.001	.088

*Note*: Reference values for impact score: normal” (score 0), “borderline” (score 1) or “abnormal” (score 2‐10). Spearman correlation: *r* = .1, weak effect; *r* = .3, medium effect; *r* = .5, strong effect. *r* values in bold letters represent significant correlations.

^a^ n = 21.

^b^ n = 35.

**TABLE 6:** Comparison of patients on and off diet revealed no significant differences of SDQ parent‐report or SDQ self‐report, neither regarding the median Total Difficulties Score nor most of the single subscales, except for *“Hyperactivity”* in adolescents

	Parent‐report			Parent‐report			Self‐report		
	2‐10 years	2‐10 years	*P*	11‐17 years	11‐17 years	*P*	11‐17 years	11‐17 years	*P*
	on diet	off diet		on diet	off diet		on diet	off diet	
	n = 17	n = 10		n = 13	n = 9		n = 13	n = 9	
	(6f, 11 m)	(6f, 4 m)		(7f, 6 m)	(4f, 5 m)		(7f, 6 m)	(4f, 5 m)	
Total Difficulties Score; Median [IQR]	10.0 [7.5; 14.0]	5.5 [4.0; 10.0]	.076	7.0 [3.5; 7.0]	8.0 [4.0; 10.5]	.311	8.0 [5.5; 12.0]	9.0 [7.5; 16.0]	.251
Emotional Problems	2.0 [0.0; 4.0]	0.5 [0.0; 2.0]	.375	2.0 [0.0;3.5]	2.0 [1.0;2.5]	.880	3.0 [1.0; 4.0]	2.0 [1.0; 4.5]	.881
Hyperactivity	4.0 [3.0;5.5]	3.0 [1.0; 4.3]	.097	**2.0** [0.5; 3.0]	**5.0** [1.5; 5.0]	**.047**	2.0 [1.0; 4.0]	4.0 [3.0; 6.0]	.057
Conduct Problems	3.0 [2.0; 4.0]	1.5 [0.0; 3.3]	.237	1.0 [0.0; 2.0]	0.0 [0.0; 1.0]	.178	1.0 [0.0; 3.0]	1.0 [0.0; 3.0]	.728
Peer Problems	1.0 [0.0; 2.0]	1.0 [0.0; 2.3]	.895	1.0 [0.0; 2.0]	0.0 [0.0; 3.5]	.970	2.0 [1.0; 3.0]	2.0 [1.0; 3.5]	.599

*Note*: *P values in bold numbers represent significant differences *P* < .05, for better visualization the corresponding results are also displayed in bold numbers, Mann‐Whitney‐*U*‐test.

No influence of metabolic control on Total Difficulties Score and no correlation between Phe concentrations and Total Difficulties Score could be detected (Table 2).

## 4 DISCUSSION

As a chronic disease, PKU and the resulting daily implementation of the lifelong dietary treatment challenge everyday life of the affected children and their families. Quality of life and psychological wellbeing of these patients are therefore often jeopardized.^2^, ^3^, ^26^ The study presented here aimed to evaluate psychological well‐being of early and continuously treated children and adolescents with PKU. SDQ self‐ and parent‐report were used to assess psychological strengths and behavioral difficulties of these patients.^19^, ^20^ Median Total Difficulties Score and median scores of subscales were within the normal range in all patients. This was true for the self‐ as well as the parent‐report. No differences between patients and controls could be found in median Total Difficulties Score. However, regarding SDQ subscales, some differences between parent‐ and self‐report as well as between patients with PKU and controls occurred. All SDQ scores correlated positively with the Impact Score, emphasizing the relevance of the results for everyday life. Overall, the children with PKU rated themselves more self‐critical than their parents. Although the group of investigated PKU patients is small and results are gathered by a single treatment center and, therefore, some limitations in data interpretation must be considered, the results provide insight into a so far little investigated field.

A chronic disease is generally associated with a higher risk for psychological impairment.^27^ Already children and adolescents with chronic diseases more often suffer from depression, anxiety disorders, and disturbed emotional or behavioral functioning.^28^, ^29^ In patients with PKU, low self‐esteem, attentional problems, lack of autonomy, depressed mood, and general anxiety occur more frequently compared to metabolically healthy peers.^8^, ^30^, ^31^ So far, there are only few systematic studies on psychological health in PKU.^4^, ^9^, ^32^ Due to recent developments in dietary therapy and implementation of the first medical treatments in PKU, these studies might be out of date.^10^, ^11^ However, patients with PKU still require a life‐long therapy and long‐term good metabolic control can only be reached by continuously high therapy adherence. Poor metabolic control negatively impacts psychological well‐being and neuropsychiatric outcome. Psychiatric problems, in turn, can lead to poorer therapy adherence and worsen metabolic control. Therefore, evaluation of psychological health of patients with PKU is of great interest. We compared patients on and off diet, but interestingly could not find significant differences between them, neither in the SDQ parent‐report nor the SDQ self‐report. However, therapy‐specific effects on well‐being cannot be differentiated with the SDQ. A specific questionnaire addressing this issue, analogous to the available specific questionnaire on quality of life in PKU, would have been preferable,^2^, ^33^ but does not exist as yet.

When comparing patients with PKU in general with their healthy peers, some abnormalities, strengths and differences could be detected. Irrespective of suffering from PKU or not, 2‐ to 10‐year‐old children showed "Conduct Problems" with a borderline score in over 50% of the cases. The "Conduct Problems" seem not to be PKU specific, but rather the items of this subscale, such as “*Often has temper tantrums or hot tempers*,” “*steals*,” can partly be attributed to age‐related development. The subjects with borderline score in this subscale were predominantly of pre‐school age. During this age, children discover their own selves, explore their influences, strive for independence, and test limits.^34^ Of notice, the parents of the investigated PKU patients reported “*stealing things at home, school or outside*” disproportionately high compared to healthy peers. Due to the strict limitation of protein rich foods within the PKU diet, parents might evaluate the behavior of their children differently compared to parents of metabolically healthy children. In this context, unfortunate snacking of protein rich and, therefore, unsuitable foods by a child with PKU might be interpreted as stealing.

In contrast, "Prosocial Behavior" appears to be a strength of 2‐ to 10‐year‐old children with PKU compared to healthy peers. Likewise, adolescent boys with PKU showed a significantly lower score for "Conduct Problems". This is in accordance with a former study investigating the personality of 58 early and continuously treated 10‐year‐old children with PKU.^35^ These patients reported significantly less masculine attitudes, but higher social commitment and complacence compared to healthy controls. Masculine attitudes include, for example, higher level of sensation seeking associated with a greater propensity for risk taking play behavior.^36^ The well‐trained social behavior might be caused by early training of empathy due to own experience with a chronic disease.

The overall perception of adolescence by the investigated parents showed that they were less critical in comparison to their children. The median Total Difficulties Score was lower compared to the self‐report, irrespective whether the adolescent suffered from PKU or not. Despite this difference might be of more statistical than practical significance, as the respective median scores were within the normal range, for girls with PKU, this difference was significant. Nevertheless, "Emotional" and "Peer Problems" were the main focus for the parents of the adolescent girls, again irrespective of suffering from PKU or not. Of note, while none of the healthy adolescent girls rated themselves as abnormal for "Emotional Problems", this was the case in almost a third of the adolescent girls with PKU. None of the adolescent boys reported Emotional Problems. Several studies already showed a higher risk for "Emotional Problems" in girls compared to their male counterparts.^37^, ^38^, ^39^ This might be a consequence of socialization effects by peers, which vary according to gender and age.^37^, ^40^, ^41^ Behavior, experience of stress, and coping strategies in dealing with peers are gender‐specific. Girls more often show cooperative, prosocial behavior, empathy, and tend to communicate their own feelings. Their socially oriented goals and behaviors may encourage concerns about social recognition, loneliness or the well‐being of a friend.^37^ In addition, young women perceive more stress than boys, especially during puberty.^40^, ^42^, ^43^, ^44^ The reported "Emotional Problems" in adolescent girls were, therefore, not surprising. Indeed, more than a quarter of the adolescent girls with PKU rated themselves to be “abnormal.” This was significantly different compared to girls from the control group. Puberty is a very vulnerable period in the life of girls with PKU, when they encounter the issue of maternal PKU syndrome for the first time. Maternal PKU syndrome is a severe embryopathy caused by high maternal Phe concentrations during pregnancy.^45^ Females with PKU who wish to have children must reintroduce the strict diet to reach low blood Phe concentration as required during infancy.^1^ This contradicts the relaxation of the diet after the age of 10 years when the nervous system is fully developed and higher blood Phe concentrations are tolerated without negative impact, and such repeated change in strategy may well lead to inner conflicts. A former study investigating health‐related quality of life in patients with PKU already showed the highest impact of PKU on the score measuring anxiety regarding blood Phe levels during pregnancy.^2^

In total, data interpretation remains difficult as the investigated cohort is small due to the fact that PKU is a rare disease and multicenter longitudinal investigations would be an important next step. However, the fact that the differences compared to the healthy population are slim but relevant could be shown in different former trials for related areas as health‐related quality of life or life satisfaction.^2^, ^45^

## 5 CONCLUSION

Overall, patients with PKU showed a SDQ Total Difficulties Score within the normal range without significant differences to healthy peers. Differences within single subscales, for example, "Emotional Problems" or "Conduct Problems", however, require separate consideration and evaluation. The SDQ is a suitable instrument to investigate patients at different stages of life. Interpretation of the revealed data should respect the special situation of patients under low‐protein diet. Implementation of the SDQ in the treatment routine, for example, at milestones in life (such as start of Kindergarten, primary school, secondary school, before transition) is conceivable. This allows early detection of emotional or social problems and supply of individual support. In this regard, special attention should be paid to adolescent PKU girls who seem to be at risk to develop emotional problems.

## CONFLICTS OF INTEREST

The authors have no conflicts of interest relevant to this article to disclose.

## AUTHORS CONTRIBUTION

A. G. Thiele: Conceptualization and study design, patient recruitment, data analysis, writing the manuscript. S. Beblo: Conceptualization and study design, patient recruitment, data analysis, writing the manuscript. N. Spieß: Assisted in conceptualization and study design of, patient recruitment, data collection, data analysis, manuscript review. R. Ascherl: Assistance in data interpretation and data analysis, statistical advice. C. Rohde: Patient recruitment, data interpretation, manuscript review. W. Kiess: Assistance in conceptualization, study design and data interpretation, manuscript review. M. Arelin: Patient recruitment, data interpretation, manuscript review. All authors approved the final manuscript as submitted and agree to be accountable for all aspects of the work.

## ETHICS STATEMENT

The study was approved by the University of Leipzig's ethics committee (registration number: 440‐12‐17 122 012).

## Data Availability

The datasets generated for this study are available on request to the corresponding author.
